# Mirror Writing and a Dissociative Identity Disorder

**DOI:** 10.1155/2009/814292

**Published:** 2009-10-26

**Authors:** Catherine Le, Joyce Smith, Lewis Cohen

**Affiliations:** ^1^Department of Neurology at the University of California, Davis, 4860 Y Street, Sacramento, CA 95817, USA; ^2^Psychiatric Consultation Service, Baystate Medical Center, 759 Chestnut Street, Springfield, MA 01199, USA

## Abstract

Individuals with dissociative identity disorder (DID) have been known to show varied skills and talents as they change from one dissociative state to another. For example, case reports have described people who have changed their handedness or have spoken foreign languages during their dissociative states. During an interview with a patient with DID, a surprising talent emerged when she wrote a sentence for the Folstein Mini-Mental State Exam—mirror writing. It is not known whether her mirror writing had a deeper level of meaning; however, it does emphasize the idiosyncratic nature of dissociative identity disorder.

## 1. Manuscript

Since the days of Leonardo da Vinci's own mirror writing, this form of penmanship has been a curious manipulation of the written word. It is defined as script that “runs in the opposite direction to normal, with individual letters reversed, so that it is most easily read using a mirror [1].” Mirror writers are often intrinsically left handed and mirror write with their left hands and may come from cultures, such as Chinese or Japanese, that normally write from right to left [1]. Mirror writing may also be found among those who have degenerative brain diseases or who have suffered brain lesions, head injuries or vitamin deficiencies [1, 2]. With practice healthy adults may also learn to mirror write. Even those with amnestic disorders can learn to mirror write after enough practice [2]. Despite these characteristics, mirror writing can also appear in the most unexpected places and from the most unexpected people, such as during a Folstein Mini-Mental State Exam with a patient.

## 2. Case Report

**Figure 1:**
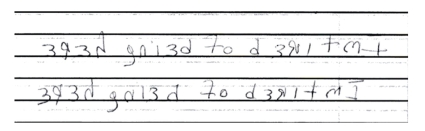
Sentence written by Ms. A.

**Figure 2:**
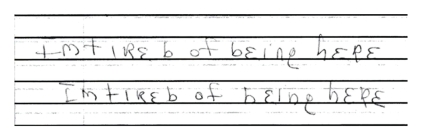
Mirror image of sentence.

Ms. A, a 46-year-old woman, was admitted for global amnesia. She was awake and alert in the emergency room but was confused about her identity, past events in the last 2 days, and how she had arrived at the hospital. A head CT scan showed no acute disease. The psychiatry team was consulted, and it was discovered that Ms. A had a previous diagnosis of dissociative identity disorder (DID). Further investigation revealed that her family recently experienced enormous stress, which may have precipitated the current episode. During the psychiatric exam, she calmly sat with her eyes closed and insisted that they were already open when asked to open them. She also repeatedly fluctuated between referring to herself as “I” or “she.” When asked to write a complete sentence during the Folstein Mini-Mental State Exam, Ms. A took the pen with her right hand and effortlessly wrote, from the right margin to the left, the mirror image of “I’m tired of being here.” When asked to write the sentence again, she wrote the same sentence in the same direction as fluidly as she had done before (Figures 1 and 2). She later reported that she was normally right handed. Ms. A ultimately received a 24/30 on the Mini-Mental State Exam, with points deducted in orientation to place and time and in short-term memory. The amnesia began to clear toward the evening, and Ms. A agreed to continue her care at a local community hospital.

People with severe DID are known to fluctuate in various abilities, “such as fluency in a foreign language or athletic abilities [3, 4].” In some case studies, people with DID are able to switch handedness [5]. For example, one case study described a 27-year-old right handed woman who would regress into a left handed child [5]. One other case study described a left handed woman with a previous history of migraines and sensory and perceptive disturbances who mirror wrote with her right hand [6]. An exhaustive literature search was unable to yield reports of people with DID who spontaneously mirror wrote during their dissociative states, thereby making Ms. A's situation distinctive. It is not known why Ms. A began mirror writing as she did in her state. As is true for Leonardo da Vinci's mirror writing, one cannot help but wonder if there was a more profound level of meaning or purpose behind such reversed script.
